# Vibroacoustic therapy in the treatment of patients with COVID-19 complicated by respiratory failure: a pilot randomized controlled trial

**DOI:** 10.3389/fmed.2023.1225384

**Published:** 2023-12-14

**Authors:** Aidos Konkayev, Assema Bekniyazova

**Affiliations:** ^1^Department of Anesthesiology and Intensive Care, Astana Medical University, Astana, Kazakhstan; ^2^The National Scientific Center of Traumatology and Orthopedics Named After Academician Batpenov N.D., Astana, Kazakhstan

**Keywords:** COVID-19, vibroacoustic therapy, respiratory insufficiency, physiotherapy, pilot randomized trial

## Abstract

**Introduction:**

Coronavirus infection is a dangerous airborne disease that can lead to serious lung damage. Data on the effectiveness of low-frequency chest vibrations in the treatment of lung diseases are available; however, not so many of them exist. Vibroacoustic pulmonary therapy is a component of physiotherapy that improves lung perfusion and drainage without requiring active patient participation. This study aimed to increase statistical efficiency through maximizing the relevant information obtained from the clinical data. Calculating the sample size to determine the power of subsequent studies was also necessary.

**Research methods:**

A pilot randomized parallel trial involving 60 patients was conducted. The patients were divided into two equal groups, where they received sessions of vibroacoustic pulmonary therapy using the “VibroLung” device in two modes “acute respiratory distress syndrome (ARDS)” and “Pneumonia,” with identical treatment. The patients were > 18 years old with detected COVID-19 by PCR and grade 2 and 3 lung lesions detected by computer tomography (CT). Blood sampling was performed in the morning at the same time before and after the hardware massage to determine PaO_2_, PaCO_2_, and P/F.

**Results:**

As a result of the test, the following data were obtained: on the first day in the group using the “ARDS” mode, PaO_2_ indicators averaged 65, CI 95% [58.6–73.2] and on average 77.5, CI 95% [69.8–85.2], “before” and “after,” respectively, which indicates improved oxygenation after the procedure. However, in the second group with the “Pneumonia” mode after its use, PaCO_2_ was higher after the session, on average 48.7, CI 95% [40.8–56.6], whereas before that, the following indicators had, on average 43.6, CI 95% [37.2–50].

**Conclusion:**

Thus, the data obtained yielded ambiguous results, which are the basis for further study in future randomized controlled trials. As the treatment of coronavirus infection has no etiological treatment, even small shifts in the therapy of this category of patients can be significant.

**Clinical trial registration:**

ClinicalTrials.gov, identifier NCT05143372.

## Introduction

COVID-19 is a highly contagious infectious disease that has had a significant impact worldwide since 2019. COVID-19 continues to affect the human body without requiring any special treatment (1, 2). In addition to the general symptoms of intoxication, the disease course varies from mild-to-severe damage to the lungs and other body systems. The treatment of patients with coronavirus pneumonia requires an adequate comprehensive approach, including physiotherapy (3, 4). The symptoms of this pathology include cough, shortness of breath, and sputum discharge (5). Vibroacoustic lung therapy can be a rational component of physiotherapy in the treatment of respiratory diseases, which, instead of manual massage, saves the time and effort of medical personnel who already have a heavy load (6). This improves perfusion and drainage functions, ultimately improving respiratory activity. Vibroacoustic therapy is of interest in many areas of medicine, there are many questions about the effective parameters of the technique (frequency, amplitude, etc.). The range of applications of vibroacoustic therapy (VAT) is wide, including the treatment of pain, fibromyalgia, neurological pathologies. A preliminary review of the treatment of pain with the use of VAT has shown a positive effect, but more reliable studies and results in this area are needed (7).

A review of 7 studies of patients with cerebral palsy showed a positive effect on motor function when using VAT (8).

In addition, there are studies in favor of low-frequency vibration (0.5–120 Hz) in patients with acute stroke with thrombolysis, dissolution of blood clots, which is important for patients with coronavirus infection (9–11).

The “VibroLung” device was used as a component of physiotherapy during the pilot trial in patients with respiratory diseases and is actively used in hospitals in the Commonwealth of Independent States (CIS) countries (6).

The purpose of this pilot test was to determine the acceptability of vibroacoustic massage.

Information on the topic was unavailable when searching the MEDLINE and Cochrane databases. In the past, when treating a patient with a coronavirus infection and concomitant background, an integrated approach using a vibroacoustic device has shown effectiveness and interest in continuing trials (12).

## Materials and methods

The study is a simple, blind, randomized parallel trial. Data were collected from the intensive care unit of the City Infectious Diseases Hospital of Astana, Republic of Kazakhstan, from December 2021 to July 2022.

Inclusion criteria:

• Age > 18 years,
• Confirmed COVID-19 using PCR test,
• The degree of lung damage according to computed tomography: CT-2 (25–50% of lung parenchyma is affected); CT-3 (50–75% of lung parenchyma is affected).

Exclusion criteria:

• Age < 18 years,
• Acute stroke,
• Acute coronary syndrome,
• Traumatic chest injury,
• Infectious processes in the chest area.

Consent to participate in this project was obtained from the participants or their guardians before the intervention. The trial included 60 patients (Supplementary material) diagnosed with pneumonia caused by coronavirus infection, confirmed using computed tomography and PCR tests for the presence of the virus. All the described patients were on a mechanically ventilator. They were divided into two equal groups. In one group, patients underwent vibroacoustic pulmonary therapy using the “VibroLung” device in a mode called “acute respiratory distress syndrome (ARDS)” (main group *n* = 30), in the other—in the “Pneumonia” mode (comparison group *n* = 30). In the “ARDS” mode, the physical emphasis is evenly distributed on slow and fast modulations with a frequency of 20 to 300 Hz, which provides both endobronchial resonance and parenchymal effects, but, as is typical for nosology and hyperhydration of the lungs in ARDS, such effects are most effective. In the “Pneumonia” mode, the effect of fast and slow modulations is applied evenly over all frequencies, aimed at draining a multi-caliber tracheobronchial tree–within 120–300 Hz (13).

Both groups had an identical algorithm of action: sessions were conducted six times a day for 5 min for 3 days in combination with treatment in accordance with the protocol for the treatment of patients with coronavirus infection of the Ministry of Health of the Republic of Kazakhstan (14). The effectiveness of the effect increases with an increase in the multiplicity, but not with an increase in the duration of the procedure (session). Effective duration of the procedure: 3–5 min (15).

The emitters of the device were applied to the most affected areas of the patient’s lungs, corresponding to the CT or radiography data, and one or the other mode of the device was turned on. Because the device has long cords for emitters and is portable, the patient’s position does not matter and does not require active participation, which is important for patients undergoing artificial lung ventilation. Immediately before the procedure, arterial blood was collected once in the morning to determine PaO_2_, PaCO_2_, and P/F blood, as well as 10 min after the session.

The initial patient data will be shown below (Table 1).

**TABLE 1:** Initial demographic data of patients.

Characteristic	Main group (“ARDS” *n* = 30)	Control group (“Pneumonia” *n* = 30)
Age, years, median [IQR]	69 [5.75]	66.5 [21]
Gender, male, *n* (%)	8 (27%)	11 (37%)
Gender, female *n* (%)	22 (73%)	19 (63%)
APACHE II, median [IQR]	10.5 [5.25]	10 [5]
PADUA, median [IQR]	7 [1.25]	6 [1]
qSOFA, median [IQR]	2 [1]	1.5 [1]
CRP, m [SD]	129.9 [82.5]	111 [71.6]
IL-6, m [SD]	177 [257.5]	392 [1077]
ESR, m [SD]	26 [14.8]	30.8 [17.1]

IQR, interquartile range; m, median; n, number; SD, standard deviation.

All data were entered into the MC Excel database as patients were registered by the researcher. At the end of the set period, the data were statistically processed.

The sample size was not calculated owing to the uneven admission of patients with coronavirus infection at different times from 2021 to 2022 because of the variability of the peak incidence.

Participants were randomly assigned to either the treatment or comparison group at a 1:1 ratio using a random number generator on the site www.randomizer.org. The researcher handed the medical workers a preprepared opaque envelope with randomly assigned numbers. Owing to the need to select a mode on the device display screen, all medical personnel knew regarding the selected mode when it was hidden from the patient.

Medical workers trained in the procedure and use of the device conducted a session of vibroacoustic therapy, as well as performed blood sampling.

Data were analyzed using the Statistical Package for Social Sciences (SPSS) version 20.

The Kolmogorov–Smirnov criterion was used to determine the normality of data. Student’s *t*-tests were used only for normally distributed data. In cases with an abnormal distribution, intragroup analysis was performed using the Wilcoxon test in each group before and after VALT.

### Ethical approval

The study was approved by the Ethics Committee of the Research Institute of Orthopedics and Traumatology in Astana on 19.11.21 and registered on ClinicalTrials.gov., ID: NCT05143372.

## Results

Regarding the initial data of patients in both groups, age and sex did not show large differences, as did scores on the APACHE II, qSOFA, and PADUA scales. Based on laboratory data, patients showed a difference in the mean values (SD) of CRP, IL-6, and ECR (Table 1).

When analyzing the results, the following information was obtained: in the group using the “ARDS” mode, on the first day, the average value of PaO_2_ increased by 12.5 mmHg. After the procedure, the median increased by 7.8 mmHg, 95% confidence interval: it became [69.8–85.2], and the standard deviation after that was 20.6 mmHg (Table 2).

**TABLE 2:** Statistical data on the application of the “ARDS” of the VALT mode before and after.

Arterial blood gases	1 day	2 day	3 day
PaO_2_ before	*Mean 65, m 66.7, CI 95% [58.6–73.2], SD 19.5*	Mean 73.2, m 73.7, CI 95% [67.3–79.2], SD 16	Mean 70.9, m 73.6, CI 95% [64.1–77.7], SD 18.2
PaO_2_ after	*Mean 77.5, m 74.5, CI 95% [69.8–85.2], SD 20.6*	Mean 74.4, m 76.3, CI 95% [67.8–80.9], SD 17.6	Mean 75.4, m 75.7, CI 95% [65.8–85], SD 25.7
PaCO_2_ before	Mean 42.7, m 36.8, CI 95% [36.4–48.9], SD 16.8	Mean 46, m 40.8, CI 95% [40.2–51.9], SD 15.7	Mean 43.6, m 37.7, CI 95% [37.2–50], SD 17
PaCO_2_ after	Mean 40.4, m 37.7, CI 95% [35.8–45], SD 12.3	Mean 48.8, m 40.4, CI 95% [40.6–57], SD 22	Mean 48.7, m 39.6, CI 95% [40.8–56.6], SD 21.1
P/F before	Mean 88.8, m 79.5, CI 95% [74.8–107.5], SD 43.8	Mean 90.6, m 83, CI 95% [80–101.4], SD 29.1	Mean 92, m 84.5, CI 95% [78–106], SD 37.6
P/F after	Mean 98.8, m 88.3, CI 95% [84.7–112.9], SD 37.8	Mean 90.6, m 87.3, CI 95% [80–101.2], SD 28.4	Mean 93.5, m 87, CI 95% [80–106.8], SD 35.7

The average value, median, 95% confidence interval, standard deviation are indicated. Statistically significant data are highlighted in italics. IQR, interquartile range; m, median; n, number; SD, standard deviation.

As for the group using the “Pneumonia” mode, the results on the change in the PaCO_2_ level on the third day were shown and were “Before” average 43.6, m 37.6, CI 95% [37.2–50], SD 17 and “After” mean 48.7, m 39.6, CI 95% [40.8–56.6] SD 21.1. Vibroacoustic pulmonological apparatus was used in this mode (Table 3).

**TABLE 3:** Statistical data when applying “Pneumonia” of the VALT mode before and after.

Arterial blood gases	1 day	2 day	3 day
PaO_2_ before	Mean 67.6, m 66.7, CI 95% [61.3–74], SD 17	Mean 71, m 64, CI 95% [58.2–83.9], SD 34.4	Mean 72, m 65, CI 95% [62.7–81.2], SD 25
PaO_2_ after	Mean 72, m 61.9, CI 95% [59.3–84.9], SD 34.4	Mean 72, m 59.3, CI 95% [61–83], SD 29.5	Mean 72.3, m 62, CI 95% [62.3–82.2], SD 26.6
PaCO_2_ before	Mean 43, m 39, CI 95% [37–48.2],SD 14.1	Mean 46, m 40.8, CI 95% [40.2–51.9], SD 15.7	*Mean 43.6, m 37.6, CI 95% [37.2–50], SD 17*
PaCO_2_ after	Mean 47.5, m 39.4, CI 95% [40.6–54.5], SD 18.6	Mean 48.8 m 40.4, CI 95% [40.6–57], SD 22	*Mean 48.7, m 39.6, CI 95% [40.8–56.6] SD 21.1*
P/F before	Mean 88.8, m 97, CI 95% [76.2–101.3], SD 33.5	Mean 89.7, m 83.7, CI 95% [75.2–104.2], SD 38.7	Mean 90, m 90, CI 95% [73.8–107.7], SD 45.4
P/F after	Mean 95.2, m 85.2, CI 95% [76.1–114.2], SD 33.5	Mean 89.9, m 74.4, CI 95% [73.7–106], SD 43.3	Mean 92, m 83.2, CI 95% [75.7–108.5], SD 43.9

The average value, median, 95% confidence interval, standard deviation are indicated. Statistically significant data are highlighted in italics. IQR, interquartile range; m, median; n, number; SD, standard deviation.

## Discussion

The purpose of this study was to determine the acceptability of vibroacoustic pulmonary massage. Taking into account the results obtained, it is necessary to note a certain positive effect of vibroacoustic lung therapy in the treatment of pneumonia in both modes of operation of the device. It should also be noted that in the “ARDS” mode, an improvement in the oxygen content in arterial blood was observed on the first day, whereas in the “Pneumonia” mode, a decrease in carbon dioxide was observed on the third day of receiving VALT sessions. These results are encouraging, but for greater reliability of the results, further study of the field of physiotherapy methods using a larger sample in a randomized controlled trial is necessary. It depends on the power, frequency or other mechanisms of action of the shaft, which have yet to be studied in more depth.

Coronavirus infection differs from other diseases with an extremely high mortality rate worldwide, including in our region, which limits the long-term results of this study.

Another disadvantage is the need to conduct the study in a single clinic. Thus, establishing a causal relationship between the observed changes is difficult.

For a more specific and accurate generalization of the results of the effects of vibroacoustic lung therapy, continuing the study with long-term follow-up is necessary, as the use of vaccines against the virus has reduced the severity of the disease and reduced the mortality of patients (16). Therefore, a multicenter study with a larger sample size is required. COVID-19 has caused enormous harm to the health of the population worldwide, and today, although not in such quantities, cases of the disease remain being registered. Therefore, owing to the lack of etiological treatment, a rational integrated approach is advisable for this category of patients (17, 18). A number of studies have focused on the positive impact and safety of using low-frequency vibrations and vibrations separately on respiratory function and on the body as a whole; however, no combination of them in one device that we attempted to study, including vibration in coronavirus disease exists (19, 20).

Vibroacoustic waves in the form of triangular-shaped modulations with a relatively “slow” period (1.1–10 s) are used for exposure in order to improve ventilation-perfusion ratios, recruitment and drainage of large-caliber bronchi. Vibroacoustic waves of “faster” modulation (0.2–1.0 s) are used to further improve the drainage function of small-caliber bronchi and sawtooth-shaped alveolar sacs with decreasing and increasing frequency. Due to the acoustic properties of the chest, the rapidly decreasing frequency of vibroacoustic waves causes an effect resembling a light tapping on the chest, the rapidly increasing frequency of vibroacoustic waves resembles the effect of soft pressure on the chest, which contributes to the discharge of sputum. According to clinical observations, the frequency range from 20 to 60 Hz most effectively affects the chest. At the same time, the frequency range of vibroacoustic waves of 37–42 Hz for most patients is the most effective in terms of creating maximum pressure fluctuations in the lumen of the large airways (21). To enhance the effect in this frequency segment, a temporary emphasis is placed in all programs. Exposure at higher frequencies is less prolonged and has less time emphasis, because when exposed at higher frequencies, the penetrating power of the sound wave is less. Thus, for effective exposure, the best effect is achieved when exposed to a relatively wide frequency range, regardless of the nature of the pathology. Low-frequency pressure fluctuations of 20–60 Hz (22) in the lumen of the respiratory tract contribute to both a better intake of air into the lungs and drainage function. Low-frequency sound waves probably have an effect on the parietal parenchyma, and the rapidly changing frequency of the signal contributes to a better drainage effect of the small bronchi. The more intense the inflammatory process, the more edematous the pulmonary parenchyma is, respectively, it is more accessible to the penetration of higher frequency sound waves (above 100 Hz). For the most optimal effect in various lung pathologies, executive programs can be more divided into 3 groups, depending on the percentage of elements of fast (with a period of 0.2–1 s) and deep slow (1.1–10 s) frequency modulation, which mainly determines the drainage effect. In pathology, where the drainage effect is not a priority (restrictive pathology), the content of such elements is less. In executive programs that pursue the main goal of improving drainage, the content of such elements is greater. The above information is according to the manufacturer’s manual.

It is worth noting the undesirable effect observed by a medical professional that when using the “ARDS” mode, vibration has greater power and possibly less comfort than when using the “Pneumonia” mode. The device “Vibrolung” was developed by a domestic manufacturer to improve the course and outcomes of respiratory diseases of various etiologies. According to the developer’s instructions, the principle of the shaft effect is based on the generation of an audio signal from a bifocal position by two emitters in the range of 20–300 Hz. The “floating effect” of the sound wave causes a resonant effect, which has a safe and highly effective effect on any kind of lesion of both the lung parenchyma itself and bronchial tree, alveoli, and vessels. The “floating” frequency of the signal allows you to achieve better results with less intensity and exposure time. The resulting vibroacoustic wave causes fluctuations, first of all, of the inhomogeneous parenchyma of the lung and intrapulmonary compartment (edema, mucus, infiltration, hypostatic transudate), which improves drainage, ventilation, and aeration and reduces penetration and spread. With mechanical breathing (ventilator), regular vibroacoustic action reduces the zone of lung collapse and atelectasis due to external pressure, which increases the positive end expiratory pressure (PEEP) without an additional increase in ventilation parameters.

The “ARDS” and “Pneumonia” modes in accordance with the manufacturer’s instructions, differ in indications for use. In this regard, distinctive properties of these schemes when used in patients with coronavirus infection, which has not been done before, have been of interest.

Researchers from CIS countries actively use this component of physiotherapy and publish data on successful results in patients with respiratory pathologies; however, no data on COVID-19 exist (7). Other researchers have reflected on the safety of using oscillator devices in human patients with COVID-19 on artificial lung ventilation (23).

According to the instructions for the device, low-frequency waves and vibrations propagating together cause vibrations of the structural units of the pulmonary parenchyma, thereby improving the perfusion and drainage function of the lungs. The formation of secretions in patients with coronavirus infection occurs in one-third of the cases (24).

As the device is relatively new and not sufficiently widespread, the data do not depend on the effectiveness of its use. Evidence for the positive effect of early physiotherapy on outcomes in patients with respiratory diseases is sufficient, but not in those with coronavirus infection (24–26). Vibration provides rapid evacuation of bronchial secretions, accelerates the gravitational redistribution of intra-pulmonary fluid, and improves ventilation–perfusion ratios (4).

Today, in the period after coronavirus infection, its impact on the respiratory system, rehabilitation, and in this case, vibroacoustic pulmonary therapy is of great importance. New lung physiotherapy devices and infections raise an immeasurable number of questions that require to be addressed.

## Conclusion

Thus, the use of vibroacoustic massage in the complex therapy of respiratory diseases may restore bronchial conduction disorders, improve lung ventilation and gas exchange, and improve blood circulation (27–30). Consequently, this can have an impact on faster clinical recovery, reducing the duration of inpatient treatment. Therefore, further multicenter randomized controlled trials are required to confirm the effectiveness of vibroacoustic therapy in this new group of critically ill patients. Moreover, to prove its effectiveness in further studies, introducing this component of physiotherapy for coronavirus infection complicated by respiratory insufficiency into clinical practice for future research in the field of rehabilitation of respiratory disorders after COVID-19 is advisable.

## Author’s note

The authors have read the CONSORT 2010 statement: extension to randomized pilot and feasibility trials, and the manuscript was prepared and revised according to the CONSORT 2010 statement: extension to randomized pilot and feasibility trials.

## Data availability statement

The original contributions presented in this study are included in this article/Supplementary material, further inquiries can be directed to the corresponding author.

## Ethics statement

The study was approved by the Ethics Committee the National Scientific Center of Traumatology and Orthopedics Named After Batpenov in Astana on 19.11.2021. Written informed consent from patients (guardians) was received.

## Authors contributions

AK: conceptualization. AB: review and editing of manuscript. Both authors issued final approval for the version to be submitted.
