# Supplementary material for: Vibroacoustic therapy in the treatment of patients with COVID-19 complicated by respiratory failure: a pilot randomized controlled trial
Source: Front Med (Lausanne). 2023 Dec 14;10:1225384. doi: 10.3389/fmed.2023.1225384 (PMC10753020; doi:10.3389/fmed.2023.1225384)

***Supplementary Material***

**Vibroacoustic therapy in the treatment of patients with COVID-19 complicated by respiratory failure: a pilot randomized controlled trial**

A. Konkayev, MD, PhD^1,2*^, A. Bekniyazova MD^1,2^.

^1^Astana Medical University, Astana city, Kazakhstan.

^2^The National Scientific Center of Traumatology and Orthopedics named after Batpenov N.D., Department of Anesthesiology and Intensive Care, Astana, Kazakhstan.

*** Correspondence:**
 A.K. Konkayev

[Konkaev19@gmail.com](mailto:Konkaev19@gmail.com)

**CONSORT 2010 Flow Diagram**


Supplement: Supplementary file 1 [file Data_Sheet_1.docx]
